# Five-day outcome of hepatitis E-induced acute liver failure in the ICU

**DOI:** 10.1186/s43066-021-00098-4

**Published:** 2021-05-21

**Authors:** Debashis Chowdhury, Farhana Mahmood, Cathryn Edwards, Simon D. Taylor-Robinson

**Affiliations:** 1 Department of Gastroenterology and Hepatology, Chattogram Maa O Shishu Hospital (CMOSH) Medical College, Chattogram, Bangladesh; 2 Department of Medicine, Chattogram Maa O Shishu Hospital (CMOSH) Medical College, Chattogram, Bangladesh; 3 Office of the President, British Society of Gastroenterology, St Andrew’s Place, London, UK; 4 Department of Surgery and Cancer, Imperial College London, St Mary’s Hospital Campus, London, UK

**Keywords:** Hepatitis E, Acute liver failure, Intensive care unit, Hepatic encephalopathy, Mannitol

## Abstract

**Background:**

Hepatitis E virus (HEV) is an important cause of acute liver failure (ALF) in Bangladesh with pregnant mothers being more vulnerable. As HEV occurs in epidemics, it limits medical capabilities in this resource-poor country. Cerebral oedema, resulting in raised intracranial pressure (ICP), is an important cause of morbidity and mortality. Practical treatments are currently few.

- To study the baseline characteristics and clinical outcome of HEV-induced ALF in a recent HEV epidemic
- To detect raised ICP clinically and observe response to mannitol infusion.

This was a prospective cohort study from June until August 2018 of 20 patients admitted to the intensive care unit (ICU) of a major Bangladeshi Referral Hospital with HEV-induced ALF. We diagnosed HEV infection by detecting serum anti-HEV IgM antibody. All were negative for hepatitis B surface antigen and hepatitis A IgM antibody. Data were collected on 5-day outcome after admission to ICU, monitoring all patients for signs of raised ICP. An intravenous bolus of 20% mannitol was administered at a single time point to patients with raised ICP.

**Results:**

Twenty patients were included in the study. Ten (50%) patients, seven (70%) females, received mannitol infusion. HE worsened in eight (40%): seven female and three pregnant. Glasgow Coma scores deteriorated in six (30%): all (100%) females and three pregnant. Consciousness status was not significantly different between pregnant and non-pregnant subjects, nor between those who received mannitol and those who did not. Six patients met King’s College Criteria for liver transplantation.

**Conclusions:**

Female patients had a worse outcome, but pregnancy status was not an additional risk factor in our cohort. Mannitol infusion was also not associated with a significant difference in outcome.

## Background

Every year, more than 20 million people contract hepatitis E virus (HEV) infection, and among them, more than 70,000 die from its complications, including acute liver failure (ALF) [1]. Most of these deaths occur in Asia, Africa and Latin America, where contaminated water from flooding of low-lying areas gives rise to epidemic of HEV infection almost every year [2].

HEV infection is common in Bangladesh; Sheikh and colleagues found IgM antibodies against HEV in 63.6% of patients with ALF, 83.3% of HBV carriers and in 7.3% of apparently healthy persons in their study [3]. According to another Bangladeshi study, in an epidemic of HEV infection, which affected more than 4000 people, most deaths occurred in pregnant women [4]. Moreover, women were more likely to die from the illness than male subjects. In a different study, HEV-induced ALF was found to result in mortality in more than 75% of subjects in their second and the third trimester of pregnancy in Bangladesh [5].

Studies in Bangladesh and India have demonstrated that cerebral oedema resulting in raised intracranial pressure (ICP) is an important cause of morbidity and mortality in HEV-induced ALF [6–8]. Mannitol infusion is often chosen as the first option [9–11] followed by hypertonic saline and moderate hypothermia to manage raised ICP in ALF patients [12, 13]. Barbiturates, indomethacin and hyperventilation were also used as short-term salvage therapies in some refractory cases [10, 11, 14]. Mannitol acts by changing the viscoelasticity of blood and drawing fluid along an osmotic gradient from brain to blood [15]. A previous study [16] suggested that infusion of mannitol could reduce the water content and volume of normal brain tissue. However, mannitol cannot act once brain tissue is permanently damaged. Canalese and colleagues in a controlled trial demonstrated that mannitol can both decrease the raised ICP level and improve survival in ALF patients [9].

Acharya and co-workers in India found that outcome of ALF in pregnant subjects was similar to that in non-pregnant subjects, and one-third of ALF patients in their study survived with aggressive conservative management [6]. The authors also observed that two-thirds of deaths due to ALF occurred within 72 h of hospital admission. Shalimar and colleagues in their study observed that the model for end-stage liver disease (MELD), ALF study group model, and King’s College Hospital criteria failed to predict outcome in HEV-induced ALF [8].

We did this study during an HEV epidemic in a major Bangladesh referral centre to audit treatment given to patients with HEV-induced ALF and to assess the effectiveness of intensive care support in the epidemic. We did this by using the clinical tools available in this resource-poor country:

- Examining the baseline characteristics and clinical outcome of HEV-induced ALF at 5 days
- Detecting raised ICP clinically and observing response to mannitol infusion, a key treatment given for HEV-associated cerebral oedema in Bangladeshi patients with ALF

## Methods

This was a prospective cohort study on baseline characteristics and clinical outcomes of 20 patients admitted to the intensive care unit (ICU) of Chattogram Maa-O-Shishu Hospital (CMOSH) in Chattogram (Chittagong), Bangladesh, with hepatitis E-induced ALF during an epidemic from June 2018 until August 2018 [17].

ALF was diagnosed by clinical detection of encephalopathy and raised INR within 24 weeks of development of jaundice [18, 19]. ALF was diagnosed by increased INR with or without hepatic encephalopathy (HE). We took a history of onset, duration and clinical features from family members of subjects and consent from the next of kin before enrolment in the study.

HE grade was defined by West Haven Criteria as follows: grade 1, any alteration in mentation; grade 2, somnolent or obtunded, but easily rousable or presence of asterixis; grade 3, rousable with difficulty; and grade 4, unresponsive to deep pain [20, 21].

We diagnosed HEV by serum anti-HEV IgM antibody [22, 23]. All patients tested negative for hepatitis B surface antigen (HBsAg) and hepatitis A (anti-HAV) IgM antibody. Other causes of acute liver failure were excluded.

We selectively intubated subjects with Glasgow Coma Scale (GCS) ≤8 and/or respiratory acidosis in the setting of severe HE (encephalopathy grade ≥ 3) [24].

We collected data regarding treatment, monitoring and hemodynamic and laboratory values daily for the first 5 days after admission to ICU. We monitored all patients for oxygenation, vital signs and raised ICP. A diagnosis of raised ICP was made when neurological examination revealed either decerebrate posture or two of the following four criteria: (1) hypertension (supine blood pressure > 150/90 mmHg), (2) bradycardia (pulse rate < 10/min for the expected pulse rate for the given body temperature), (3) pupillary changes and (4) neurogenic hyperventilation (hyperventilation in the absence of metabolic or respiratory cause) [25].

We infused an intravenous bolus of 20% mannitol in ten patients with a clinical diagnosis of raised ICP. We corrected any metabolic derangement before mannitol infusion, and all patients received standard anti-coma and supportive measures [14, 26]. Only single infusions of mannitol were administered to those requiring treatment—the dose was not repeated in any patient at any time point.

We performed statistical analysis using the STATA software, version 13 (StataCorp, College Station, TX, USA), expressing continuous variables as mean, standard error, and confidence intervals, with categorical variables as frequencies and percentages. We compared continuous data using two-sample Wilcoxon rank-sum (Mann-Whitney) test and categorical data using Fisher’s exact test. A *p* value of <0.05 was considered significant. We defined outcomes as HE deterioration and deterioration of GCS after 5-day stay in the ICU.

## Results

**Fig. 1:**
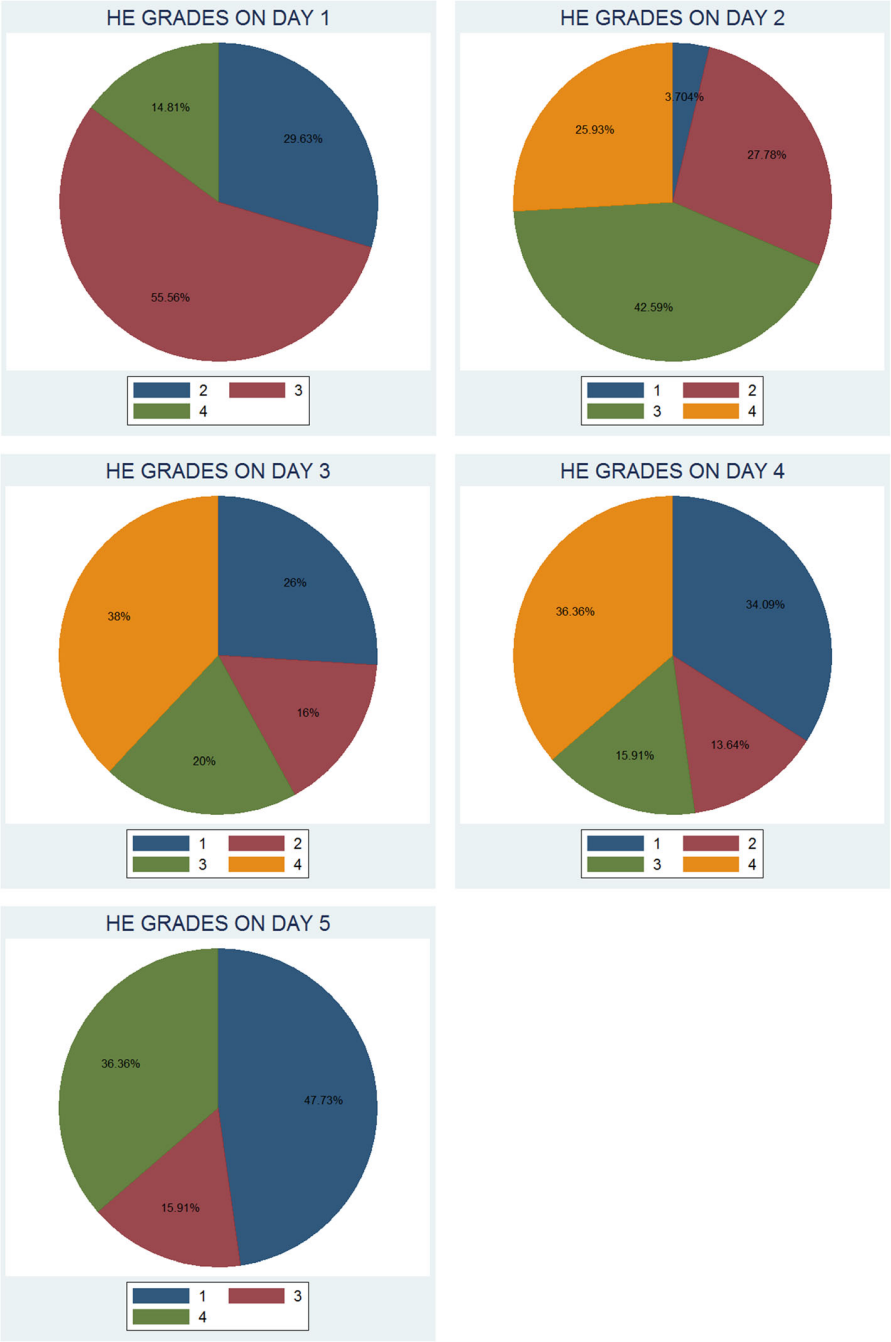
Hepatic encephalopathy grades from day 1 to day 5

**Fig. 2:**
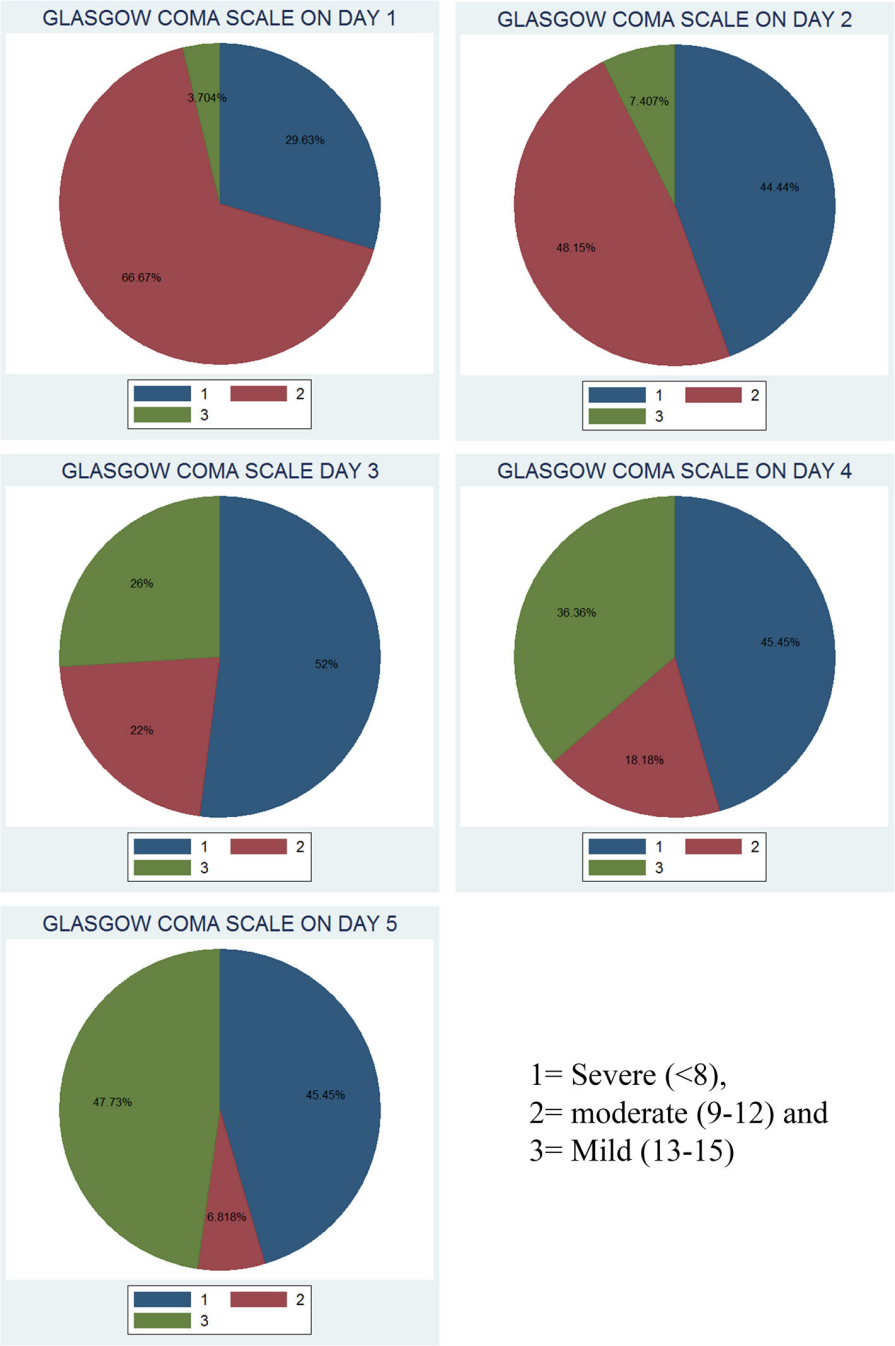
Glasgow Coma Scale from day 1 to day 5

**Fig. 3:**
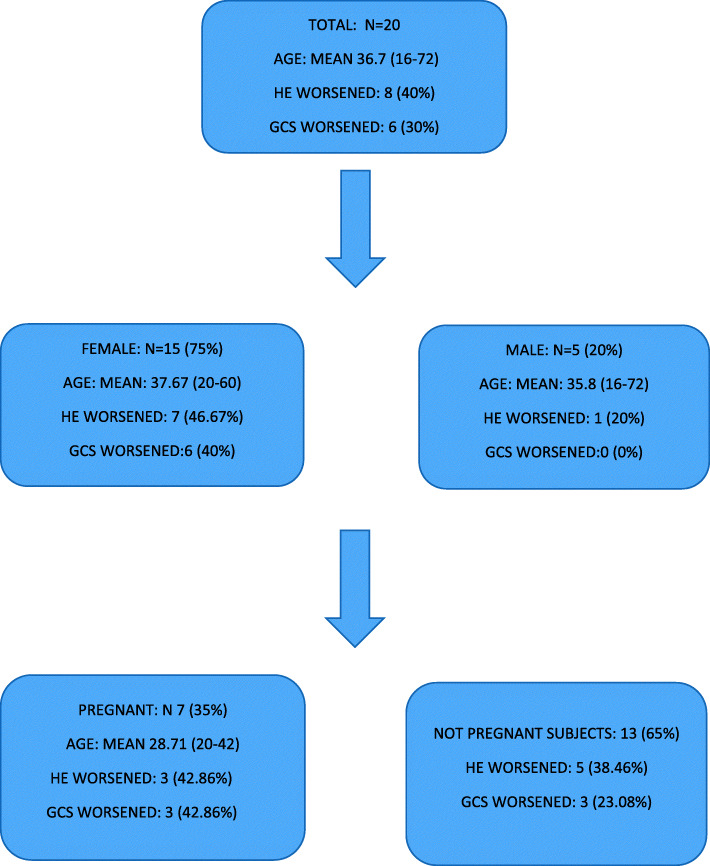
Flow chart of the patient journey

A total of 20 subjects were included in the study. Table 1 shows the demographic and biochemical characteristics of our subjects. Figures 1 and 2 show HE grades from day 1 to day 5 and GCS scores from day 1 to day 5, respectively. Figure 3 shows a flow chart of the patient journey.

**Table 1:** Demographic and biochemical characteristics

Variable	Mean	Standard error	[95% Conf. Interval]	
Age (years)	36.7	3.23	29.92	43.48
Serum Bilirubin mg/dL	10.87	1.04	8.69	13.06
Serum ALT Units/L	1528	210	1087	1970
MELD Score	29.95	2.39	24.95	34.95
INR on Day 1	2.83	0.40	1.98	3.68
INR on Day 2	3.02	0.47	2.04	3.99
INR on Day 3	2.89	0.43	1.98	3.80
INR on Day 4	2.81	0.42	1.92	3.71
INR on Day 5	3.01	0.44	2.07	3.94
Serum Creatinine On Day 1 (mg/dL)	2.45	0.52	1.37	3.53
Serum Creatinine On Day 2 (mg/dL)	2.49	0.49	1.46	3.51
Serum Creatinine On Day 3 (mg/dL)	2.64	0.55	1.48	3.80
Serum Creatinine On Day 4 (mg/dL)	2.28	0.46	1.31	3.25
Serum Creatinine On Day 5 (mg/dL)	2.36	0.46	1.38	3.35

HE worsened in eight subjects (40%); seven among them (87.5%) were female, and of these, three (37.5%) were pregnant individuals. GCS deteriorated in six (30%); of these, all (100%) were females, and of those, three (50%) were pregnant.

The majority (75%) among our subjects were female. Seven (46.67%) out of 15 females and one (20%) out of five male subjects developed HE deterioration. HE deterioration was not significantly different between male and female subjects (Fisher’s exact=0.603).

Six (40%) out of 15 female subjects and none (0%) out of five male subjects developed deterioration of GCS. Deterioration of GCS was not significantly different between male and female subjects (Fisher’s exact = 0.260).

Seven (35%) out of 20 subjects were pregnant. Three (42.86%) out of seven pregnant subjects and five (38.46%) out of 13 non-pregnant subjects developed HE deterioration. HE deterioration was not statistically significantly different between pregnant and non-pregnant subjects (Fisher’s exact=1.000). Three (42.86%) out of seven pregnant subjects and three (23.08%) out of 13 non-pregnant subjects developed deterioration of GCS. Deterioration of GCS grades was not significantly different between pregnant and not pregnant individuals (Fisher’s exact=0.613).

Six (30%) out of 20 subjects met King’s College Criteria for liver transplantation. Six (40%) out of 15 female and none (0%) out of five male subjects met King’s College Criteria for liver transplantation (*p*=0.260). Three (42.86%) out of seven pregnant subjects and three (23.08%) out of 13 non-pregnant subjects met King’s College Criteria for liver transplantation (Fisher’s exact = 0.613). Four (66.67%) among these six subjects developed HE deterioration (*p*=0.018), and five (83.33%) developed deterioration of GCS (Fisher’s exact=0.002).

One subject, a female, 30 years old, pregnant, serum bilirubin 17 mg/dL, ALT 3120 units/L, MELD score 45, HE grade IV, GCS <8, INR 4.12 and creatinine 5.1 mg/dL, died on the third day in the ICU.

### Mannitol infusion requirements

Ten (50%) out of 20 subjects received mannitol infusion. Two patients (both female and one being pregnant) with raised ICP could not be given mannitol infusion because of renal impairment (serum creatinine more than 4.5 mg/dL). Seven (46.67%) out of 15 female and three (60%) out of five male subjects received a mannitol infusion. Three (42.86%) out of seven pregnant subjects received a mannitol infusion.

HE deteriorated in 2 (10%) out of 10 patients who received and 6 (30%) out of 10 patients who did not receive mannitol. HE deterioration was not significantly different between patients who received and those who did not receive mannitol (1-sided Fisher’s exact = 0.085).

GCS deteriorated in 1 (5%) out of 10 patients that received a mannitol infusion and 5 (25%) out of 10 patients who did not receive a mannitol infusion. Deterioration of GCS was also not significantly different between subjects who received mannitol and those who did not receive mannitol (1-sided Fisher’s exact = 0.070).

### Mechanical ventilation requirement

Seven (35%) out of 20 subjects: three out of five males, four out of 15 females and of these, two out of seven pregnant subjects, received mechanical ventilation on day 1. Nine (55%) out of 20 subjects: three out of five males and six out of 15 females and out of these, three out of seven pregnant subjects, received mechanical ventilation on day 2. On day 3, nine out of 19 subjects: two out of four male subjects and seven out of 15 female subjects and of these, three out of seven pregnant subjects, received mechanical ventilation. On day 4, seven out of 17 subjects: two out of four male subjects and five out of 13 female subjects and of these, one out of five pregnant subjects, were ventilated. On day 5, seven out of 17 subjects: two out of four male subjects and five out of 13 female subjects and of these, one out of five pregnant patients, were ventilated.

#### Vasopressor requirements

From day 1 to day 3, a total of seven (2 males, 5 females and of these, 2 pregnant) subjects received vasopressor support. On day 4 and day 5, six (2 males, 4 females and of these, 1 pregnant) subjects received vasopressor support.

### Haemodialysis requirements

Haemodialysis was started when subjects developed renal failure (urine output less than 300 mL/day; serum creatinine more than 400 micromol/L (4.52 mg/dL) with a normal central venous pressure: two subjects—a female, 22 years (creatinine 8.2 mg/dL), second, a female, 60 years (serum creatinine 7.1 mg/dL)). Both received haemodialysis for 5 days in ICU.

## Discussion

This study reports the acute outcomes in a typical resource-poor setting in Bangladesh. The majority (75%) of subjects were female. Seven (35%) patients in our study were pregnant. Three (42.86%) out of 7 pregnant subjects and 3 (23.08%) out of 13 non-pregnant subjects had worsening of HE. Deterioration of HE was not statistically significantly different between pregnant and non-pregnant subjects (Fisher’s exact = 1.000).

In a previous study on this HEV epidemic in Chattogram (Chittagong), Bangladesh, we found that among 230 of our patients 24 (10.4%) had developed ALF. Four (1.8%) among them died due to multiorgan failure with acute kidney injury, and all were pregnant [17].

Other studies in Bangladesh also found that women, especially pregnant women, were more susceptible to HEV-induced ALF and had worse outcome when they developed ALF [4, 5]. Moreover, in another Bangladeshi study, the authors observed that mortality approached 75% when women in the second and third trimester of pregnancy developed HEV-induced ALF [5].

In prospective studies comparing HEV hepatitis and non-HEV hepatitis in India, the authors observed that 55–70% of pregnant subjects with HEV hepatitis and 10–20% of pregnant subjects with non-HEV hepatitis developed liver failure [27, 28]. Furthermore, a study in China also found that 15–60% of the pregnant subjects with acute HEV hepatitis developed ALF [29].

On the other hand, in a study in India, which included 20 years’ data from a single tertiary centre, the authors observed that pregnant and non-pregnant subjects had equal chances of developing HEV-induced ALF [30]. In that study, which compared 249 (38.5%) pregnant subjects with 341 non-pregnant women and girls and 425 men and boys, aged 15 to 45 years, the authors found that the mortality rate of pregnant women and girls (53.8%) was similar to age-matched non-pregnant women and girls (57.2%), men and boys (57.9%) (*P* = 0.572). The authors also observed that the clinical and biochemical profile, disease intensity and sequelae were also comparable in those three study groups. Although a significantly higher percentage of ALF was attributable to HEV among pregnant subjects (59.4%) in comparison to both non-pregnant women and girls (30.4%) and men and boys (23.1%), (*P* < 0.001), the outcome of HEV-induced ALF had no association with the gender and pregnancy status of the subjects (*P* = 0.103). Furthermore, the mortality of pregnant subjects in HEV-induced ALF of 51% (74/145) and non-HEV-induced ALF of 54.7% (52/95) was not significant (*P* > 0.1). In addition, the authors also found that the outcome in HEV-induced ALF in pregnant subjects was not associated with the trimester of pregnancy.

Shalimar and colleagues in their study observed that the increased HEV-induced ALF and resulting mortality might be due to the increased susceptibility of women, especially pregnant women, to HEV infection during an epidemic in affected populations [31]. Moreover, the authors found that after the development of ALF, the prognosis was not dependent on pregnancy status.

According to a study in Bangladesh [7], cerebral oedema found in 48 (71.6%) among 67 subjects was the most important cause of death in the ALF patients. Acharya and co-workers in a study in India [25], which included 423 consecutive patients with ALF due to hepatotropic viruses (predominantly non-A, non-B), found that the presence of cerebral oedema was an independent predictor of adverse outcome.

We found that 12 (60%) among our 20 subjects showed clinical features of raised ICP. Ten (50%) subjects received mannitol infusion. HE deteriorated in two (10%) out of 10 subjects who received mannitol and 6 (30%) out of 10 subjects who did not receive mannitol. GCS deteriorated in one (5%) out of 10 subjects who received and 5 (25%) out of 10 subjects who did not receive mannitol. Although the outcome was not found to be statistically significant for either HE worsening (*p*= 0.085) or GCS deterioration (*p*= 0.070), the reason for this can be explained by the small sample size of the study.

Canalese and co-workers compared the effects of prophylactic dexamethasone and mannitol infusion to revert cerebral oedema in subjects with ALF with HE grade IV in a randomised controlled clinical trial. Cerebral oedema resolved significantly more frequently in 17 among 34 subjects who received mannitol and in 17 among 34 subjects who did not (*p*<0001) [9]. Survival was also found to be significantly better in subjects that received mannitol (*p* 0.008).

Acharya and colleagues [6] in India found that outcome of ALF in pregnant subjects was similar to that in non-pregnant subjects and one-third of ALF patients survived with aggressive conservative therapy. Our study also found that the outcome in pregnant and non-pregnant subject was not statistically significantly different. Moreover, HE worsened in 8 (40%) and GCS deteriorated in 6 (30%) of our subjects during the 5 days. More than 60% of our patients showed clinical improvement with conservative therapy.

Shalimar and his colleagues also observed that MELD, an ALF study group model, and King’s College Hospital criteria failed to predict outcome in HEV-induced ALF. In our study, six patients (30%) met King’s College Criteria for liver transplantation. Four (66.67%) among those 6 patients developed deterioration of their encephalopathy grades (Fisher’s exact =0.018), and 5 (83.33%) developed deterioration of GCS (Fisher’s exact=0.002) [8].

### Limitations of our study

Most of our patients were transferred from different inpatient units—medicine, obstetrics and surgery when they were diagnosed as having HEV-induced ALF. We could not take a detailed history from the patients or look for other causes which could have modified the clinical illness in our subjects owing to cerebral obtundation. Furthermore, we could not test for HEV RNA and detect the HEV genotype in our subjects, owing to resource issues.

Moreover, we diagnosed raised ICP, based on clinical parameters, which may not be optimum in some cases, again owing to lack of resources. The sensitivity and specificity of clinical parameters for the diagnosis of raised ICP in the settings of ALF are low. Neurological examination and clinically establishing raised ICP in these patients can be challenging [24].

## Conclusion

Most of our patients were female, and many among them were pregnant. Female patients developed worse outcome than male patients. Pregnancy status was not associated with worse outcome in our cohort. More than 50% of our subjects had cerebral oedema, as evidenced by clinical signs of raised ICP. Mannitol infusion, although not found to be statistically significant, was observed to improve outcome in them. Future directions should be early detection of cerebral oedema using more sophisticated imaging techniques and management of cerebral oedema using new therapeutic agents. Finally, HEV infection is common in Bangladesh, and HEV epidemics can result in significant morbidity and mortality in vulnerable populations, which include women and especially pregnant women. Aggressive conservative management with proper ICU protocols of HEV-induced ALF may save many lives.

## Data Availability

The datasets used and analysed during the current study are available from the corresponding author on request.

## Abbreviations

ALF: Acute liver failure
CMOSH: Chattogram Maa O Shishu Hospital
GCS: Glasgow Coma Scale
HE: Hepatic encephalopathy
HEV: Hepatitis E virus
ICP: Intracranial pressure
ICU: Intensive care unit
IRB: Institutional Review Board

## References

[1] Rein DB, Stevens GA, Theaker J, Wittenborn JS, Wiersma ST (2012). The global burden of hepatitis E virus genotypes 1 and 2 in 2005. Hepatology.

[2] Teshale EH, Hu DJ, Holmberg SD (2010). The two faces of hepatitis E virus. Clin Infect Dis.

[3] Sheikh A, Sugitani M, Kinukawa N, Moriyama M, Arakawa Y, Komiyama K (2002). Hepatitis E virus infection in fulminant hepatitis patients and an apparently healthy population in Bangladesh. Am J Trop Med Hyg.

[4] Gurley ES, Hossain MJ, Paul RC, Sazzad HMS, Islam MS, Parveen S (2014). Outbreak of hepatitis E in urban Bangladesh resulting in maternal and perinatal mortality. Clin Infect Dis.

[5] Krawczynski K, Hepatitis E (1993). Hepatitis E. Hepatology.

[6] Acharya SK, Panda SK, Saxena A, Gupta SD (2000). Acute hepatic failure in India: a perspective from the East. J Gastroenterol Hepatol.

[7] Alam S, Azam G, Mustafa G, Azad AK, Haque I, Gani S, Ahmad N, Alam K, Khan M (2009). Natural course of fulminant hepatic failure: the scenario in Bangladesh and the differences from the west. Saudi J Gastroenterol.

[8] Shalimar KS, Gunjan D, Sonika U, Mahapatra SJ, Nayak B, Kaur H, Acharya SK (2017). Acute liver failure due to hepatitis E virus infection is associated with better survival than other etiologies in Indian patients. Dig Dis Sci.

[9] Canalese J, Gimson AE, Davis C, Mellon PJ, Davis M, Williams R (1982). Controlled trial of dexamethasone and mannitol for the cerebral oedema of fulminant hepatic failure. Gut..

[10] Lee WM, Stravitz RT, Larson AM (2012). Introduction to the revised American Association for the Study of Liver Diseases Position Paper on acute liver failure 2011. Hepatology.

[11] Stravitz RT, Kramer AH, Davern T, Shaikh AO, Caldwell SH, Mehta RL, Blei AT, Fontana RJ, McGuire BM, Rossaro L, Smith AD, Lee WM (2007). Intensive care of patients with acute liver failure: recommendations of the U.S. Acute Liver Failure Study Group. Crit Care Med.

[12] Murphy N, Auzinger G, Bernel W, Wendon J (2004). The effect of hypertonic sodium chloride on intracranial pressure in patients with acute liver failure. Hepatology.

[13] Larsen F, Murphy N, Bernal W, Bjerring P, Hauerberg A, Wendon J (2011). The prophylactic effect of mild hypothermia to prevent brain oedema in patients with acute liver failure: results of a multicentre randomised controlled trial [Abstract]. J Hepatol.

[14] Ede RJ, Gimson AE, Bihari D, Williams R (1986). Controlled hyperventilation in the prevention of cerebral oedema in fulminant hepatic failure. J Hepatol.

[15] Bruce DA, Berman WA, Schut L (1977). Cerebrospinal fluid pressure monitoring in children. Physiology pathology and clinical usefulness. Adv Pediatr.

[16] Videen TO, Zazulia AR, Manno EM, Derdeyn CP, Adams RE, Diringer MN, Powers WJ (2001). Mannitol bolus preferentially shrinks non-infarcted brain in patients with ischemic stroke. Neurology..

[17] Biswas RS, Hasan F, Sultana A, Uddin MK, Chowdhury D, Rosy S, Mamun S (2019). A documentation of hepatitis outbreak in Chittagong. Chattagram Maa-O-Shishu Hosp Med Coll J.

[18] Lee WM, Squires RH, Nyberg SL, Doo E, Hoofnagle JH (2008). Acute liver failure: summary of a workshop. Hepatology..

[19] Tandon BN, Bernauau J, O'Grady J (1999). Recommendations of the International Association for the Study of the Liver Subcommittee on nomenclature of acute and subacute liver failure. J Gastroenterol Hepatol.

[20] Conn HO, Lieberthal MM (1979). The hepatic coma syndromes and lactulose.

[21] Atterbury CE, Maddrey WC, Conn HO (1978). Neomycin-sorbitol and lactulose in the treatment of acute portal-systemic encephalopathy. A controlled, double-blind clinical trial. Am J Dig Dis.

[22] Clayson ET, Myint KS, Snitbhan R, Vaughn DW, Innis BL, Chan L, Cheung P, Shrestha MP (1995). Viremia, fecal shedding, and IgM and IgG responses in patients with hepatitis E. J Infect Dis.

[23] Favorov MO, Fields HA, Purdy MA, Yashina TL, Aleksandrov AG, Alter MJ, Yarasheva DM, Bradley DW, Margolis HS (1992). Serologic identification of hepatitis E virus infections in epidemic and endemic settings. J Med Virol.

[24] Warrillow SJ, Bellomo R (2014). Preventing cerebral oedema in acute liver failure: the case for quadruple-H therapy. Anaesth Intensive Care.

[25] Acharya SK, Dasarathy S, Kumer TL, Sushma S, Prasanna KS, Tandon A, Sreenivas V, Nijhawan S, Panda SK, Nanda SK (1996). Fulminant hepatitis in a tropical population: clinical course, cause, and early predictors of outcome. Hepatology..

[26] Polson J, Lee WM (2005). AASLD position paper: the management of acute liver failure. Hepatology..

[27] Khuroo MS, Kamili S (2003). Aetiology and prognostic factors in acute liver failure in India. J Viral Hepat.

[28] Patra S, Kumar A, Trivedi SS, Puri M, Sarin SK (2007). Maternal and fetal outcomes in pregnant women with acute hepatitis E virus infection. Ann Intern Med.

[29] Li XM, Ma L, Yang YB, Shi ZJ, Zhou SS (2005). Clinical characteristics of fulminant hepatitis in pregnancy. World J Gastroenterol.

[30] Bhatia V, Singhal A, Panda SK, Acharya SK (2008). A 20-year single-center experience with acute liver failure during pregnancy: is the prognosis really worse?. Hepatology..

[31] Shalimar ASK (2013). Hepatitis E and acute liver failure in pregnancy. J Clin Exp Hepatol.

